# Temperature-Dependent Olive Pomace Extraction for Obtaining Bioactive Compounds Preventing the Death of Murine Cortical Neurons

**DOI:** 10.3390/ijms25020907

**Published:** 2024-01-11

**Authors:** Alessandro Alberto Casazza, Michela Capraro, Marco Pedrazzi, Giulia D’Agostino, Franco Onofri, Antonella Marte, Roberta De Tullio, Patrizia Perego, Monica Averna

**Affiliations:** 1 Department of Civil, Environmental Engineering, University of Genoa, 16145 Genova, Italy; alessandro.casazza@unige.it (A.A.C.); giulia.dagostino@edu.unige.it (G.D.); p.perego@unige.it (P.P.); 2 Department of Experimental Medicine, University of Genova, 16132 Genova, Italy; michela.capraro@edu.unige.it (M.C.); marco.pedrazzi@unige.it (M.P.); franco.onofri@unige.it (F.O.); antonella.marte@unige.it (A.M.); roberta.detullio@unige.it (R.D.T.); 3 National Research Centre for Agricultural Technologies (CN AgriTech), 80138 Naples, Italy; 4 IRCCS, Ospedale Policlinico San Martino, 16132 Genova, Italy

**Keywords:** olive pomace, antioxidant activity, HPTE, NMDAR, calcium homeostasis, murine cortical neuron

## Abstract

High-pressure and temperature extraction (HPTE) can effectively recover bioactive compounds from olive pomace (OP). HPTE extract obtained by extracting OP with ethanol and water (50:50 *v*/*v*) at 180 °C for 90 min demonstrated a pronounced ability to preserve intracellular calcium homeostasis, shielding neurons from the harmful effects induced by N-methyl-d-aspartate (NMDA) receptor (NMDAR) overactivation, such as aberrant calpain activation. In this study, the extraction temperature was changed from 37 to 180 °C, and the extracts were evaluated for their antioxidant potency and ability to preserve crucial intracellular Ca^2+^-homeostasis necessary for neuronal survival. Additionally, to verify the temperature-induced activity of the extract, further extractions on the exhausted olive pomace were conducted, aiming to identify variations in the quality and quantity of extracted phenolic molecules through HPLC analysis. The results revealed a significant increase in bioactive compounds as a function of temperature variation, reaching 6.31 ± 0.09 mgCAE/mL extract for the extraction performed at 180 °C. Subsequent extraction of the exhausted residues yielded extracts that remained active in preventing calcium-induced cell death. Moreover, despite increased antiradical power, extracts re-treated at 180 °C did not display cell protection activity. Our results indicate that the molecules able to maintain physiological Ca^2+^-homeostasis in murine cortical neurons in conditions of cytotoxic stimulation of NMDAR are wholly recovered from olive pomace only following extraction performed at 180 °C.

## 1. Introduction

Over the years, food waste management has become a topic of deep interest in the scientific community since it has been estimated that about 50–60% of solid waste produced worldwide consists of food waste [1]. Every year, more than 1.3 billion tons of food are thrown away along the entire food chain, corresponding to about a third of the global production of food for human use and more than a quarter of global agricultural production [2]. Since waste disposal is the most common method for managing food waste, this organic waste is not only the cause of an ethical problem but also responsible for a negative environmental impact [3].

The oil industry has a significant economic, social, and environmental impact on the Mediterranean countries, which are responsible for about 98% of world olive oil production [4]. However, the exponential increase in olive oil production has generated a consequent increase in waste that can be used to extract high-value-added compounds. The olive pomace is a solid by-product composed mainly of fibrous parts, such as pulp, skin, and stones, containing about 5–8% oil and a percentage of water that can vary between 25 and 70% depending on the extraction method used [5]. Due to the high quantity of phenolic compounds, olive pomace is highly polluting and phytotoxic to the environment, making it one of the most contaminating effluents in the agricultural production system [6]. However, polyphenols can be used in various fields, from nutraceutical to pharmaceutical, making olive pomace an economical source of various high-value-added compounds. These molecules can sequester or inhibit reactive oxygen and nitrogen species, reducing potential inflammatory events. At the same time, thanks to their anti-radical power, they can transfer electrons to free radicals, preventing adverse effects on the organism [7]. Different studies have highlighted the diverse biological properties of polyphenols, including anti-inflammatory, antioxidant, cardioprotective, and neuroprotective activities [8,9,10]. The precise mechanisms remain elusive despite the established protective effects of polyphenols against excitotoxicity in neuronal cells. Specifically, research indicates that certain compounds within polyphenols shield neurons by diminishing glutamate-induced Ca^2+^ influx, inhibiting Protein Kinase C activity, and subsequently preventing the phosphorylation of NR1 in the N-methyl-d-aspartate (NMDA) receptor (NMDAR) [11].

The literature reported that the extraction technique and the adopted operating conditions strongly influence the quantity and quality of extracted polyphenols, resulting in a varied impact on the polyphenols’ ability to counteract specific stress induced on endothelial cell lines [12].

Several studies report that extractions carried out at high pressures and temperatures (HPTE) without oxidizing agents lead to a significant recovery of antioxidant compounds from plant matrices, substantially reducing extraction times compared to other extraction techniques [13,14].

In this context, it has been reported that bioactive molecules contained in HPTE can mitigate calcium-induced cell damage across various cell lines [15], preventing cell death.

Moreover, Franchi et al. (2020) [16] reported that the extract obtained using HPTE (180 °C), contrary to that obtained at room temperature, can maintain intracellular calcium homeostasis, protecting neurons from the detrimental effects triggered by NMDAR overactivation, such as aberrant calpain activation [17,18]. However, in previous works [15,16], the strict temperature dependence of compounds extracted from olive pomace capable of maintaining intracellular calcium homeostasis in murine cortical neurons has yet to be clarified. Thus, given the potential applications of HPTE in preventing neuronal death, in the present study, we adopted high-pressure and high-temperature extraction to recover bioactive compounds from olive pomace (OP), varying the extraction temperature from 37 to 180 °C. In particular, we assessed the efficacy of the extracts in terms of both antioxidant power and their ability to maintain the intracellular Ca^2+^-homeostasis essential for neuronal survival following NMDAR overactivation. Furthermore, to determine the temperature-triggered activity of the extract, additional extractions on the extraction residue (exhausted olive pomace) were performed by HPLC analysis, attempting to identify the variation of extracted phenolic molecules in terms of quality and quantity.

## 2. Results and Discussion

### 2.1. Effects of Extraction Temperature on Total Polyphenol Contents and Antiradical Power

To assess the effect of temperature on the recovery of antioxidant compounds, primarily polyphenols, and to identify potential changes in the quality of the extract associated with the cracking of molecules and macromolecules (lignin) present in olive pomace, the samples were treated at different temperatures. The effect of temperature on the extraction process was then assessed regarding radical-scavenging ability through HPLC analysis and cell viability assays on both bEnd-5 cells and murine cortical neurons.

OP was extracted using an HPTE reactor at 37, 120, and 180 °C for 90 min, and the solid residues (OPR) of the three extraction processes, after the removal of the extracts by centrifugation, were further subjected to HPTE extraction at a temperature of 180 °C for the same time.

A strong correlation between the antiradical power and the extraction temperature was already observed by Aliakbarian et al. (2011) [19].

As seen in Table 1, an increase in extraction temperature from 37 to 120 °C leads to an almost 5-fold increase in the total polyphenol content in the extract. Similarly, when the temperature increases from 120 to 180 °C, the polyphenol content increases, although somewhat. The TPC increase is mainly due to the effect of temperature on the solvent’s extraction capacity. Indeed, increased temperatures reduce the viscosity of liquid solvents, enhancing the penetration of matrix particles and augmenting the extraction process. Furthermore, elevated temperatures can disrupt the strong interactions between solutes and matrices induced by van der Waals forces, hydrogen bonding, and dipole attractions. This event, in turn, exerts a considerable influence on the dynamics of the extraction process [20].

Moreover, the extraction at temperatures higher than the solvent boiling point (92 °C) increases the pressure inside the extraction vessel. The applied pressure compels the solvent to permeate regions within the matrices that would typically remain inaccessible under ambient atmospheric conditions [21]. Similarly, when the extraction temperature was changed from 37 to 120 °C and then to 180 °C, an increase in ARP was also observed, confirming that the activity of the extracted molecules was maintained despite the elevated temperature. Several authors working with operating conditions comparable to those used in this work have obtained the following results: Tapia-Quirós et al. (2020) [22], using a microwave-assisted extraction with an extraction time of 90 min and a solid-liquid ratio of 1:20, obtained a yield of TPC in olive pomace hydroalcoholic extract of about 10 mg_GAE_/g_DB_, while Belghith et al. (2023) [23], using an ultrasound-assisted extraction in an ethanol solution with a solid-liquid ratio of 1:10, gained a TPC of around 5 mg_TE_/g_DB_. Considering the TPY resulting from the sum of polyphenols recovered with OP180 and OPR180 extractions as the maximum TPY (108.32 mg_GAE_/g_DB_), an extraction efficiency of 5.4, 23.3, and 58.3% can be found for OP37, OP120, and OP180 extractions. The results change drastically for the extractions performed at 37 and 180 °C when considering the TP recovered with the second extraction performed on their respective residues. Indeed, the obtained extraction efficiencies were 60.5% and 60.9% for OP37/OPR37 and OP120/OPR120, respectively. The temperatures can explain the results obtained, which led to an improvement in mass transfer processes, resulting in a higher extraction rate and reduced extraction time. In this respect, Cacace and Mazza reported that elevated temperatures positively impacted extraction by increasing the solubility and diffusion coefficient of anthocyanins and polyphenols in general [24].

The temperature is a fundamental parameter for the extraction yield of polyphenols. Putnik et al. [25] observed that an increase in temperature of up to 80 °C can lead to an increase in TPC. Over this temperature, a reduction in TPC was observed; extended exposure to elevated temperatures can amplify reactions like hydrolysis and oxidation, leading to the degradation of thermos-sensitive polyphenols. Moreover, under high-temperature conditions, polyphenols could undergo isomerization, degradation, and polymerization reactions [26].

The significant increase in temperature and pressure, along with the presence of ethanol, can promote lignin extraction from the olive kernel. Liao et al. [27] stated that, in general, lignin extracted under conditions of relatively high temperature, extended reaction time, elevated catalyst concentration, and diluted ethanol showed increased antioxidant activity. This enhancement is attributed to a higher presence of phenolic hydroxyl groups, fewer aliphatic hydroxyl groups, a lower molecular weight, and a narrower polydispersity.

Another consideration must be made regarding the time required to reach the operating temperature, which varies depending on the set point (5, 15, and 25 min for 37, 120, and 180 °C, respectively). A similar consideration can be applied concerning the time elapsed between the end of the extraction and the attainment of the minimum temperature necessary to open the extraction chamber. These additional times could affect the antioxidant recovery/degradation. A possible solution could be found in continuous extraction. Indeed, when working in continuous mode, the effect caused by the time required to reach the operating temperature is removed [28].

**Figure 1:**
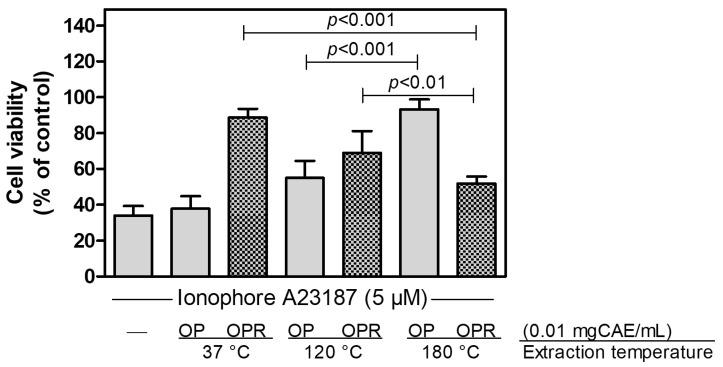
Effect of the OP extracts and of the corresponding OPR on ionophore A23187 induced bEnd5 cell death. bEND5 cells (2 × 10^4^/well) were exposed for 1 h to ionophore A23187 in the absence or in the presence of the indicated extracts. Data are means ± SD (*n* = 6 for each experimental condition). *p* < 0.001 and *p* < 0.05, according to ANOVA, followed by Tukey’s post-hoc test.

Thus, to evaluate the effect of OP extracts obtained at different temperatures on cell viability, we treated bEnd-5 cells with cytotoxic concentrations of Ca^2+^-ionophore in the absence or presence of the OP and the corresponding OPR. As reported in Figure 1, although we observed a trend in bioactive compound enrichment in parallel with temperature augment, we obtained the optimal protective effect on cells only with OP180. Consequently, both OPR37 and OPR120 were able to protect the cell viability in a way significantly different from OPR180, probably because the bioactive molecules trapped in the OPR can be recovered in a second step at 180 °C. These data indicate that only the extraction temperature of 180 °C allows obtaining molecules protecting bEnd-5 cells against death induced by an alteration in intracellular Ca^2+^-concentration.

**Figure 2:**
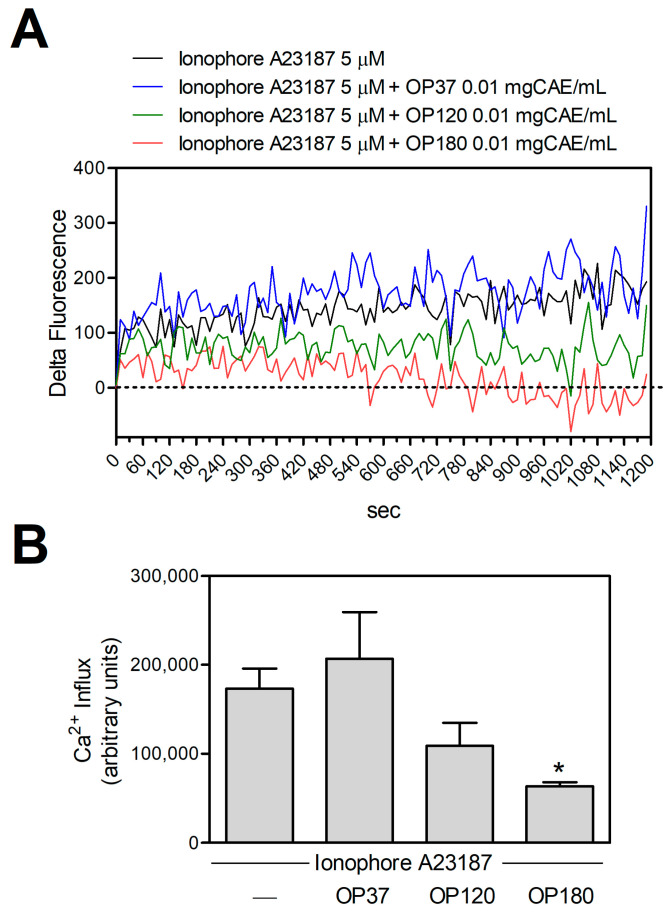
Effect of the OP extracts on [Ca^2+^]_i_ increase induced by ionophore A23187 in bEnd5 cells. (**A**) bEND5 cells (2.5 × 10^4^/well) were seeded in 96-well black plates. Calcium Green-1AM(CG)-loaded cells were treated with the indicated stimuli, and the calcium-dependent fluorescence was monitored for 20 min. (**B**) For each experimental condition, the area under the curve was quantified. Data are means ± SD (*n* = 11, 4, 8, and 7 for each condition, respectively). * *p* < 0.05, according to Kruskal–Wallis test, followed by Dunn’s post-hoc test.

It has been reported that the cell protective effect of OP180 is due to its ability to maintain the intracellular level of Ca^2+^ close to physiological concentrations [16]. To investigate this ability and the failure of the extracts obtained at temperatures under 180 °C on cell protection, we measured the intracellular Ca^2+^ concentration changes following the cell treatment with cytotoxic concentrations of Ca^2+^-ionophore in the absence or presence of OP extracts obtained at different temperatures. Figure 2A,B report that only OP180 significantly decreased the intracellular Ca^2+^-concentration. Neither OP37 nor OP120 could return the intracellular calcium concentration to physiological levels. Thus, only the temperature of 180 °C allowed the extraction of molecules, preventing cell death by maintaining intracellular calcium homeostasis.

**Figure 3:**
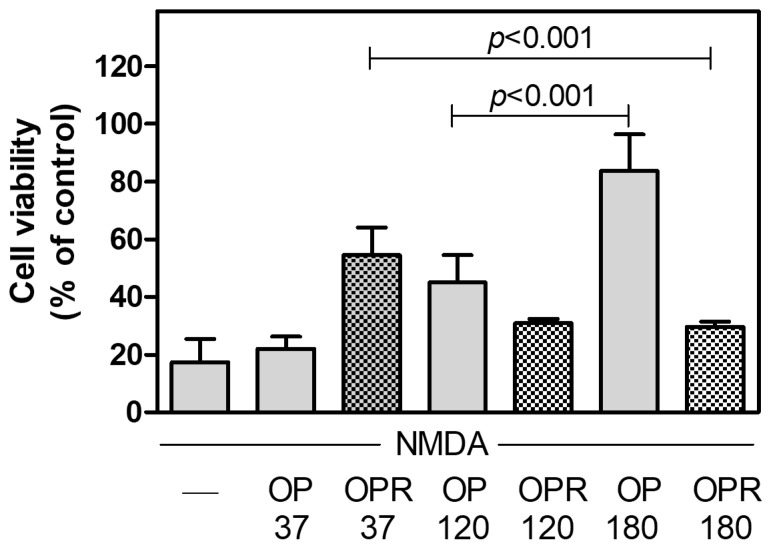
Effect of the OP extracts and of the corresponding OPR on NMDA-induced cortical neuron death. Murine cortical neurons (2.5 × 10^4^/well) were exposed for 24 h to NMDA in the absence or in the presence of the indicated extracts. Data are means ± SD (*n* = 10 for each condition). *p* < 0.001, according to ANOVA, followed by Tukey’s post-hoc test.

To further investigate the protective effect of the extracts obtained at different temperatures in conditions of calcium-mediated excitotoxicity, we used murine cortical neurons as a cellular model, as previously reported [15]. After 14 days in vitro (DIV), we exposed mature neurons to cytotoxic concentrations of NMDA in order to promote extensive cell death. As reported in Figure 3, although we observed the presence of some bioactive cell-protective compounds in OP120, which were absent in OP37, similarly to bEnd-5 cells, we obtained the optimal protective effect on cortical neurons only with OP180. Moreover, analogously to bEnd-5 cells, OPR37 protected cell viability in a way significantly different from OPR180. These results demonstrate that the molecules protecting murine cortical neurons from cytotoxic stimulation of NMDAR and trapped in the residues obtained following extraction at 37 °C (OPR37) are fully recovered only following extraction at 180 °C (OP180).

**Figure 4:**
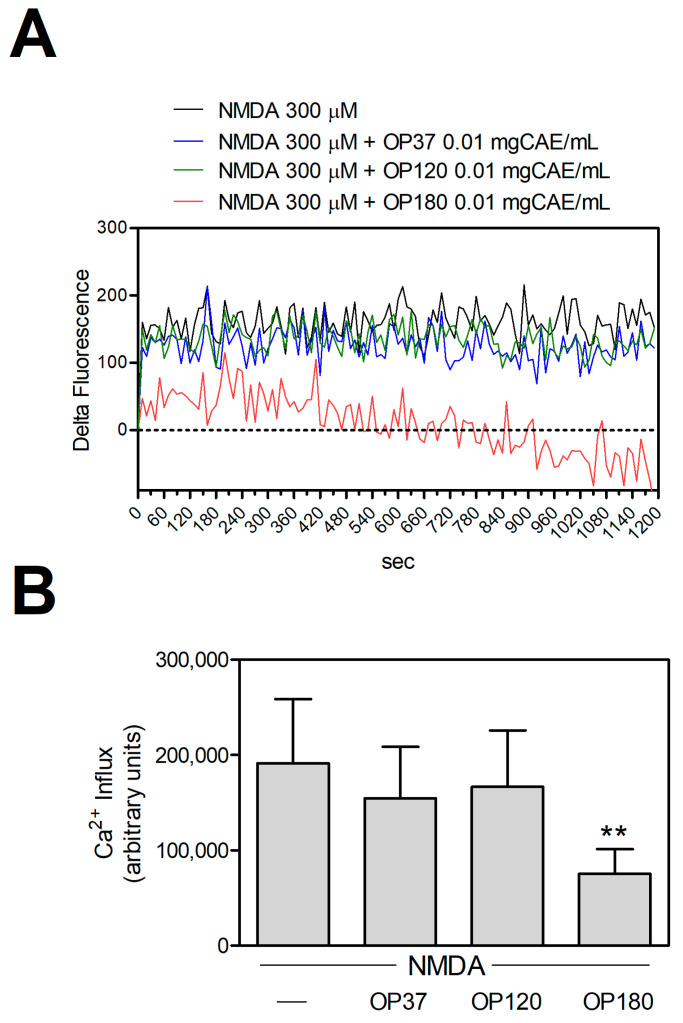
Effect of the OP extracts on [Ca^2+^]_i_ increase induced by NMDA in cortical neurons. (**A**) Murine cortical neurons (2.5 × 10^4^/well) were seeded in 96-well black plates. Calcium Green-1AM(CG)-loaded cells were treated with the indicated stimuli, and the calcium-dependent fluorescence was monitored for 20 min. (**B**) For each experimental condition, the area under the curve was quantified. Data are means ± SD (*n* = 7, 7, 10, and 8 for each condition, respectively). ** *p* < 0.01, according to ANOVA test, followed by Tuckey’s post-hoc test.

As expected, in conditions of toxic NMDAR stimulation, only OP180, but neither OP37 nor OP120, significantly reduced the consequent increase in intracellular calcium concentration (Figure 4). Thus, analogously for bEnd-5 cells, only the extraction at 180 °C allows to obtain bioactive molecules able to restore intracellular Ca^2+^ homeostasis, selectively regulating the influx of Ca^2+^ through the NMDAR.

### 2.2. Further Treatment at 180 °C of the Extracts

To dispel the doubt that molecules extracted at low temperatures may not be activated by the further step at high temperatures and pressures, we decided to submit the extracts obtained at 37, 120, and 180 °C to a second HPTE treatment at a temperature of 180 °C.

Table 2 reports the TPC values for extracts obtained at 37, 120, and 180 °C, subjected to additional treatment at 180 °C for 90 min. As can be observed, after the treatment at 180 °C, there is an increase in the total polyphenol content in the extracts obtained at 37 and 120 °C (59 and 33%, respectively), while the extract obtained at 180 °C undergoes a 13% reduction in polyphenol content. Such an increase, more pronounced in the extract at 37 °C, may be attributed to both the hydrolysis phenomena of glucosylated polyphenols into simpler polyphenols with higher activity and the chemical reactions (cracking, hydrolysis, synthesis) between macromolecules (such as proteins, cellulose, and hemicellulose) that can interact during the Folin–Ciocalteu colorimetric assay [29].

It is interesting to note the significant increase in the antioxidant power for the extract at 37 °C treated at 180 °C, showing a 3.5-fold increase compared to that of the pre-treatment residue, a value markedly higher than the mere increase in the TPC. It can thus be hypothesized that there are new molecules with significantly greater antioxidant power than those initially present, although they do not contribute to the increase in TPC. An opposite trend can be observed for the tests with OP120/180 (11% increase) and OP180/180 (21% reduction).

**Figure 5:**
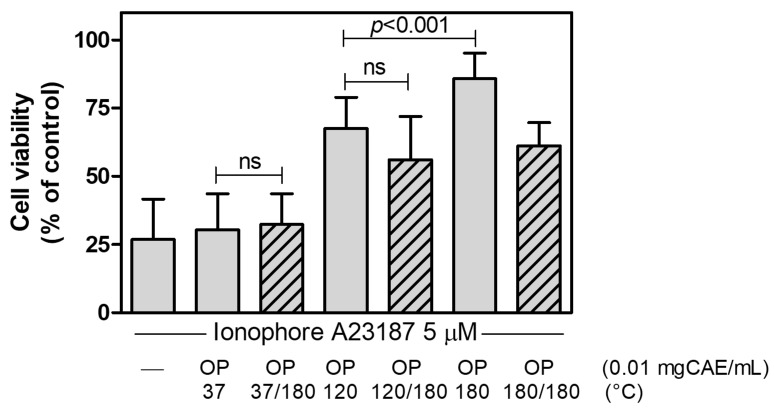
Effect of the OP extracts and of the corresponding extracts subjected to additional 180 °C-treatment on ionophore A23187-induced bEnd5 cell death. bEND5 cells (2 × 10^4^/well) were exposed for 1 h to ionophore A23187 in the absence or in the presence of OP extracts obtained at the indicated temperatures or of the corresponding extracts subjected to additional 180 °C-treatment. Data are means ± SD (*n* = 21 for each experimental condition, except *n* = 6 for OP180/180). *p* < 0.001 and ns (not statistically significant), according to ANOVA, followed by Tukey’s post-hoc test.

Despite the significant increase in the antioxidant power of OP37/180, the effect on cell viability is comparable to that of the untreated extract (OP37). Indeed, as reported in Figure 5, when we treated bEnd-5 cells with cytotoxic concentrations of Ca^2+^-ionophore in the absence or presence of OP extracts obtained at different temperatures as well as of the same extracts subjected to additional treatment at 180 °C, we observed comparable results on cell viability. These data suggest that the temperature of 180 °C can extract bioactive molecules from OP without activating molecules already extracted or that the potentially modified molecules are not present in sufficient quantities in the extract to lead to significant activity.

### 2.3. HPLC Analysis

**Figure 6:**
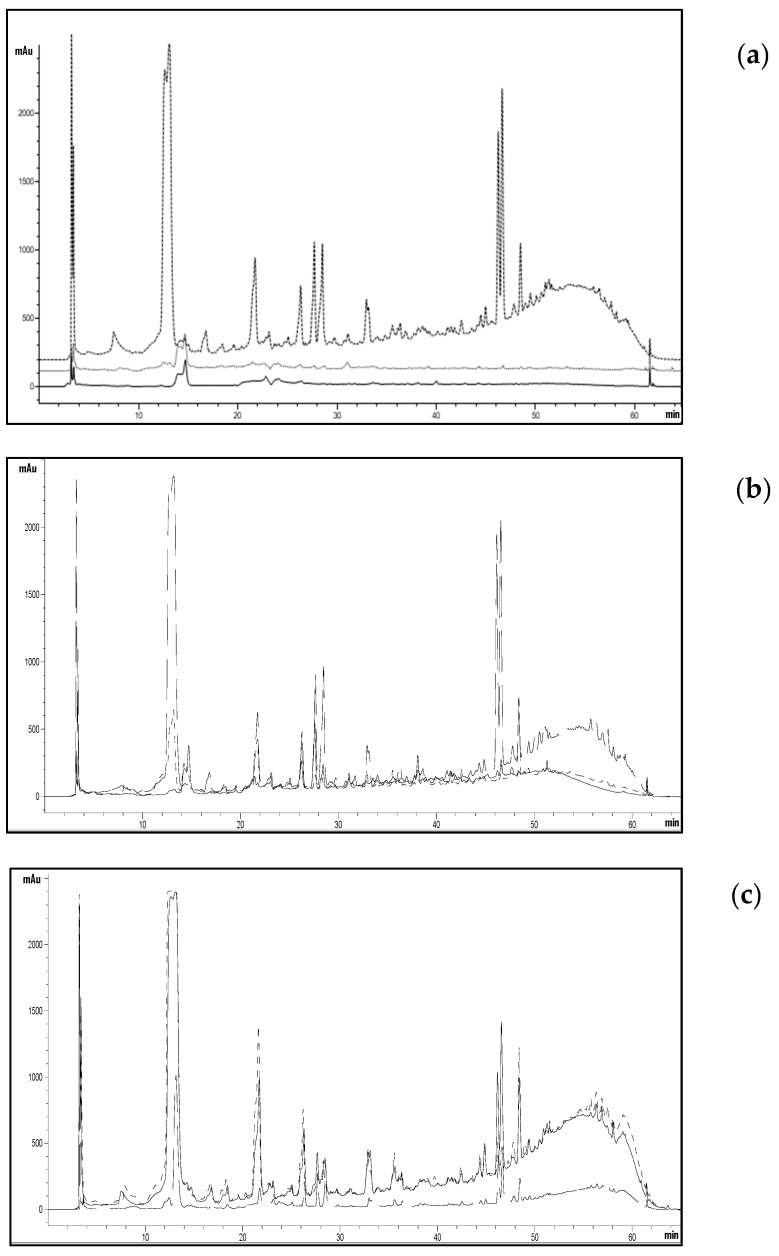
HPLC chromatograms (280 nm) of OP (----) and OPR (^____^) obtained at 37 °C (**a**), 120 °C (**b**), and 180 °C (**c**) and of OP after treatment at 180 °C (^…..^).

All the obtained extracts were analyzed using HPLC to assess how both the extraction temperature, the 180 °C treatment of the extraction residue, and an additional 180 °C treatment of the three extracts could influence the qualitative-quantitative composition of the extracts. As shown in Figure 6, a significant increase in extracted compounds is observed upon changing the extraction temperature.

Considering the extract obtained at 37 °C (Figure 6a), it can be noted that a subsequent extraction of the same does not lead to a substantial modification of the chromatogram, similar to what happens for the extracts obtained at 120 and 180 °C (Figure 6b and Figure 6c, respectively). The treatment of extracts at 180 °C appears not to substantially modify the molecules already present in the three extracts.

Table 3 reports the sums of peak areas corresponding to the observed extracts at a wavelength of 280 nm. The area significantly increases with the extraction temperature, following the trend observed for PT content and antioxidant power. If we look at the total area of the chromatograms related to the extraction residues, a remarkable increase (13 and 2.6 times, respectively) is noticeable compared to that obtained with extractions at 37 and 120 °C. Meanwhile, for OPR180, the total area is nearly five times smaller than that of OP180. This result is because extracting at 37 and 120 °C results in a partial release of extractable molecules from OP, which are recovered when the depleted matrix is treated at 180 °C. On the other hand, extraction at 180 °C leads to a massive recovery of molecules, significantly depleting the extraction residue.

The result of the analysis of the three identified polyphenols is quite interesting. While their concentration increases with the extraction temperature, a different trend is observed for 4-hydroxybenzoic acid compared to caffeic acid and oleuropein when the extracts return to 180 °C. Considering OP37, when the extract is heated to 180 °C, the percentage of 4-hydroxybenzoic acid increases from 1.3 to 16.8 mg/L, while caffeic acid and oleuropein undergo no significant variations. Considering OP120 and OP180, although lower, an increase in hydroxybenzoic acid is also observed; the other two compounds undergo degradation, with a reduction in concentration in the respective extracts submitted to the treatment at 180 °C.

The increase in 4-hydroxybenzoic acid may be due to the presence of lignin in the extract, which can undergo hydrolysis, releasing this phenolic compound, as reported by He et al. [30]. The results of the HPLC analysis suggest that, although 4-hydroxybenzoic acid cannot be the bioactive molecule able to protect neuronal cells, the effect on the cellular activity of the extracts obtained at 180 °C may be due not only to purely extractive phenomena but also to reactions that lead to the release of active molecules from macromolecules.

## 3. Materials and Methods

### 3.1. Raw Materials and Chemicals

Olive pomace (OP), obtained after a three-phase process (*Taggiasca cultivar*), was supplied by a local producer (Liguria, Italy). The fresh OP was collected and dried in a laboratory oven at 50 °C until there was constant moisture, and then stored in dark conditions at room temperature.

Ethanol, methanol, acetonitrile, acetic acid, and sodium carbonate were purchased from Carlo Erba Reagents (Cornaredo, Milan, Italy), while Folin–Ciocalteu’s reagents included caffeic acid, 2,2′-azino-bis-(3-ethylbenzothiazoline-6-sulfonic acid) diammonium salt (ABTS), potassium persulfate, 6-hydroxy-2,5,7,8-tetramethychroman-2-carboxylic acid (Trolox), ionophore A23187, NMDA, and neutral red solution from Merck-Sigma-Aldrich (Milan, Italy). Calcium Green™-1AM, Neurobasal™ medium, B-27 supplement, Glutamax^®^, penicillin, and streptomycin solution were purchased from Life Technologies Italia (Milan, Italy). DMEM, FBS, MEM non-essential amino acids 100×, and trypsin-EDTA 1× solutions were from Euroclone (Milan, Italy).

### 3.2. High-Pressure and Temperature Extraction (HPTE)

All the extractions were carried out in a laboratory-scale stainless-steel extractor in which an impeller provided homogeneous mixing. The first extraction process (90 min) was carried out using a hydroalcoholic solution (50:50 *v*/*v*) as an extractive solvent. The effect of temperature on phenolic compound recovery was investigated using three different extraction temperatures (37, 120, and 180 °C) with a solid/liquid ratio of 1:10 (*w*/*v*) (both for olive pomace and olive pomace residues) [19]. All extractions were performed under a nitrogen atmosphere. At the end of the extraction process, all the samples were centrifuged at 6000× *g* for 10 min and then filtered through a 1.2 µm paper filter.

Samples were stored at 4 °C before further analysis. Moreover, exhausted olive pomace OP obtained from HPTE extraction was dried in a laboratory oven to a constant weight and then stored at room temperature in dark conditions.

The extracts obtained, respectively, at 37, 120, and 180 °C were subjected to further treatment at 180 °C using the same extractor and extraction conditions. Dried, exhausted OP was used as the starting biomass for further extraction. In particular, each residue was treated under the same conditions as described above, but at a temperature of 180 °C. Extracts and residues from these second extractions have been treated under the same conditions as described above. All experiments were performed in triplicate.

### 3.3. Total Polyphenols and Antiradical Activity Analysis

The Folin–Ciocalteu and ABTS colorimetric assays, respectively, were used to determine the amount of total polyphenols present in the extracts and their respective antiradical activities.

All the extracts obtained by HPTE were analyzed in terms of total phenolic compound concentration using a modified version of the Folin–Ciocalteu colorimetric assay [14]: 4.8 mL of deionized water were added to 0.2 mL of appropriately diluted extract, then 0.5 mL of Folin–Ciocalteu reagent and 1.0 mL of a saturated solution of sodium carbonate were added, respectively, followed by 3.5 mL of deionized water to reach a total final volume of 10 mL. The solution was incubated at room temperature in the dark for 1 h. Then, the absorbance reading was performed in triplicate using a Lambda 25 spectrophotometer (Perkin Elmer, Wellesley, MA, USA) at a wavelength of 725 nm. A calibration curve was obtained using methanolic standard solutions of caffeic acid in a range from 0.01 to 1.00 mg/L. Total polyphenols were expressed as milligrams of caffeic acid equivalents (CAE) per milliliter of extract (mg_CAE_/mL_extract_). The method responses were described by Equation (1) (R^2^ = 0.996):

ABS_725_ = 0.0023 × TP (1)

The antiradical activity was determined using an ABTS colorimetric assay [31]. This method involves the use of [2,2-azinobis-(3-ethylbenzothiazoline-6-sulfonic acid)] radical chromophore cation (ABTS•^+^), which is obtained through the reaction between ABTS and potassium persulfate. Then, 50 µL of appropriately diluted extract were added to 1 mL of ABTS•^+^ solution, and it was incubated at room temperature in the dark for 2 min. Subsequently, the absorbance reading was performed in triplicate at a wavelength of 734 nm.

A calibration curve was obtained using a methanolic standard solution of 6-hydroxy-2,5,7,8-tetramethychroman-2-carboxylic acid (Trolox) in a range from 0 to 100 mg/L. Thus, the antiradical activity was expressed as milligrams of Trolox equivalents (TE) per milliliter of extract (mg_TE_/mL_extract_). The method’s responses were described by Equation (2) (R^2^ = 0.993):

ABS_734_ = −0.0049 × C + Abs_blank_ (2)

### 3.4. HPLC-DAD Analysis

The profile of the extracts was acquired by high-performance liquid chromatography (HPLC) (Hewlett Packard, 1260 Series, Palo Alto, CA, USA) combined with a diode array detector (DAD), equipped with a C18 reverse-phase column (Model 201TP54, Vydac, Hesperia, CA, USA), as described by Paini et al. (2016) [14].

The mobile phase consisted of water/acetic acid 99:1% *v*/*v* (solvent A) and methanol/acetonitrile 50:50% *v*/*v* (solvent B). The solvent gradient was adjusted based on the following parameters: starting from 0 to 5% solvent B in 5 min, then from 5 to 30% B in 25 min, followed by a change from 30 to 40% B in 10 min, then from 40 to 48% B in 5 min, from 48 to 70% B in 5 min, a further increase from 70 to 100% B in 5 min, and afterward isocratic at 100% B for 5 min, and finally returning to the initial conditions over 10 min, with an additional 12 min for column equilibration. The flow rate of the solvent was set at 1 mL/min. Samples, with an injection volume of 20 µL, were subjected to centrifugation at 14,000 rpm for 15 min before analysis. Detection during the analyses was conducted at wavelengths of 280. The column temperature was maintained at 30 °C throughout the process.

### 3.5. Total Solids and Bulk Density

The total solids of the extracts obtained from the HPTE process were determined gravimetrically. A total of 2 mL of samples were put in pre-weighted crucibles and then placed in a laboratory oven at 105 °C overnight. They were then cooled in a desiccator on silica gel to a constant weight. The crucible is calibrated on an analytical balance, and 2 mL of sample is added and placed in the oven overnight. It is then cooled in a desiccator on silica gel to a constant weight. The term bulk density refers to the density of a powder, considering both intra-particle and inter-particle spaces. This property was measured by loading a 10 mL cylinder with the residues obtained after the respective extractions and then weighing its contents on an analytical balance [32].

### 3.6. Cell Culture

Primary neuron cultures were prepared from the cerebral cortices of E18 0-day-old mouse embryos from Charles River Laboratories Italia s.r.l. (Lecco, Italy) and maintained as described in (Franchi et al., 2020) [16]. Neurons were allowed to grow functional and structurally mature networks before use after 14 DIV. The experimental procedures were carried out in accordance with the guidelines established by the European Communities Council (Directive 2010/63/EU of 4 March 2014) and were approved by the Italian Ministry of Health (authorization n. 22418/2020-N.REQ).

Mouse brain endothelioma (bEnd5) cells were kindly provided by L. Riboni (Department of Medical Chemistry, Biochemistry, and Biotechnology, University of Milan, LITA-Segrate, Milan, Italy) and maintained in continuous culture at 37 °C (5% CO_2_) with DMEM growth medium containing 10% fetal bovine serum, 10 units·mL^−1^ penicillin, 100 μg mL^−1^ streptomycin, 2 mM l-glutamine, 1 mM sodium pyruvate, and supplemented by MEM non-essential amino acids solution.

### 3.7. Cell Viability Assay

Cortical neurons or bEnd5 cells (seeded in a 96-well microplate at 2.5 × 10^4^/well) were exposed to 300 µM NMDA or 5 µM ionophore A23187, respectively, in the absence or presence of 0.01 mg_CAE_/mL of OP extracts, obtained at different temperatures. After a specific time of incubation (24 h for neurons and 1 h for the cell line) at 37 °C in a 5% CO_2_ humidified atmosphere, cell viability was measured using the NRU assay, as reported by Repetto et al., 2008 [33].

### 3.8. [Ca^2+^]_i_ Assay

Cortical neurons or bEnd5 cells (seeded in a 96-well microplate at 2.5 × 10^4^/well) were incubated in HEPES buffer (NaCl 128 mM, KCl 2.4 mM, MgSO_4_ 1.2 mM, CaCl_2_ 1.2 mM, KH_2_PO_4_ 1.2 mM, glucose 10 mM, and HEPES 10 mM, pH 7.3–7.4) containing 10 µM Calcium Green™-1AM. After 30 min at 37 °C, cells were washed twice with HEPES buffer and then exposed to 5 µM ionophore A23187 (bEnd5) or 300 µM NMDA (neuron) in the absence or presence of 0.01 mg_CAE_/mL of OP extracts, obtained at different temperatures, at 37 °C for 20 min. CG-dependent fluorescence (excitation 485 nm and emission 535 nm) was monitored every 10 s using the top reading mode in the fluorescence multilabel reader LB 940 Mithras (Berthold Technologies, Baden Württemberg, Germany). [Ca^2+^]_i_ increase is expressed as “Delta Fluorescence”, which is the difference between the CG-dependent fluorescence of the stimulated samples and the ones of the vehicle-treated samples, both measured at each recording time and subtracted by the one measured at the starting time. To estimate the calcium influx in each experimental condition, the area underlying the curve was measured by means of the Prism 4.02 software package (GraphPad Software, San Diego, CA, USA).

### 3.9. Statistical Analysis

Data were presented as mean ± SD. The data distribution was analyzed by the Kolmogorov–Smirnov test. The significance of the difference was analyzed by ANOVA, followed by a post hoc Tukey’s test or Kruskal–Wallis test, followed by Dunn’s post-hoc test, using the Prism 4.02 software package, with statistical significance taken at *p* < 0.05, as indicated in the figure legends.

## 4. Conclusions

In conclusion, the data obtained demonstrate that the molecules protecting murine cortical neurons from death in conditions of cytotoxic stimulation of NMDAR are wholly recovered from OP only following extraction at 180 °C. Moreover, treatment at 180 °C is required to extract bioactive molecules from OP but not activate molecules already extracted. Considering the potential applications of OP180 in preventing neuronal death, the relevance of this detailed characterization of the olive pomace extract is obtaining the necessary information to narrow the focus on the molecule responsible for the neuroprotective activity. In this context, since our results suggest the presence of lignin in the extract, future activity will be dedicated to treating the lignocellulosic component of olive pomace (kernel) to assess whether the treatment at 180 °C leads to the release of molecules with the desired activity. Finally, the data obtained will be compared with those obtained by treating other lignocellulosic matrices and commercial lignin.

## Figures and Tables

**Table 1:** Total polyphenol concentration (TPC), Total polyphenol yield (TPY), Antiradical power (ARP), and Extract total solids of the olive pomace (OP) extracts obtained at 37, 120, and 180 °C (OP37, OP120, and OP180) and of the olive pomace residue extracts obtained at 180 °C (OPR37, OPR120, and OPR180).

	Extraction Temperature (°C)	TPC (mg_CAE_/mL_extract_)	TPY (mg_CAE_/g_OP_)	ARP (mg_TE_/mL_extract_)	Extract Total Solids (mg/mL)	Residue Bulk Density (g/cm^3^)
OP37	37	0.58 ± 0.01	5.80 ± 0.15	0.68 ± 0.05	5.48 ± 0.67	0.502 ± 0.018
OP120	120	2.25 ± 0.22	25.22 ± 2.17	5.39 ± 0.05	11.53 ± 0.23	0.388 ± 0.018
OP180	180	6.31 ± 0.09	63.13 ± 0.90	15.06 ± 0.06	30.88 ± 0.06	0.319 ± 0.013
OPR37	180	5.98 ± 0.40	59.78 ± 4.00	9.43 ± 0.35	30.53 ± 1.07	0.239 ± 0.012
OPR120	180	4.07 ± 0.13	40.74 ± 1.29	9.16 ± 0.07	30.22 ± 0.55	0.262 ± 0.029
OPR180	180	3.42 ± 0.08	45.20 ± 0.84	4.63 ± 0.29	12.55 ± 0.49	0.232 ± 0.002

**Table 2:** Total polyphenol concentration (TPC), Antiradical power (ARP), and Extract total solids of the olive pomace (OP) extracts obtained at 37, 120, and 180 °C were followed by re-treatment at 180 °C (OP37/180, OP120/180, and OP180/180).

	TPC (mg_CAE_/mL_extract_)	ARP (mg_TE_/mL_extract_)	Extract Total Solids (mg/mL)
OP37/180	0.92 ± 0.06	3.21 ± 0.36	12.55 ± 0.49
OP120/180	3.00 ± 0.05	5.99 ± 0.42	2.90 ± 0.40
OP180/180	5.48 ± 0.73	11.94 ± 0.32	8.15 ± 0.85

**Table 3:** The total area of HPLC peaks observed at 280 nm and concentration (mg/L) of three selected polyphenols of olive pomace (OP) extracts obtained at 37, 120, and 180 °C (OP37, OP120, and OP180) of the olive pomace residue extracts obtained at 180 °C (OPR37, OPR120, and OPR180) and of OP extracts obtained followed by re-treatment at 180 °C (OP37/180, OP120/180, and OP180/180).

	Total Area_280nm_	4-Hydroxy Benzoic Acid (mg/L)	Caffeic Acid (mg/L)	Oleuropein (mg/L)
OP37	70,514	1.3	16.8	53.7
OP120	312,870	16.8	32.1	1107.9
OP180	1,070,876	32.2	41.3	897.4
OPR37	915,217	28.2	48.8	1038.7
OPR120	814,442	20.4	34.6	877.8
OPR180	219,365	5.6	15.5	122.5
OP37/180	104,862	16.8	17.3	54.0
OP120/180	359,021	29.2	28.7	439.4
OP180/180	1,159,472	44.4	17.6	571.5

## Data Availability

The data presented in this study are available on request from the corresponding author.
